# Subgingival Microbiome Colonization and Cytokine Production during Early Dental Implant Healing

**DOI:** 10.1128/mSphereDirect.00527-17

**Published:** 2017-11-29

**Authors:** Jeffrey B. Payne, Paul G. Johnson, Car Reen Kok, João C. Gomes-Neto, Amanda E. Ramer-Tait, Marian J. Schmid, Robert W. Hutkins

**Affiliations:** a Department of Surgical Specialties, College of Dentistry, University of Nebraska Medical Center, Lincoln, Nebraska, USA; b Department of Internal Medicine, College of Medicine, University of Nebraska Medical Center, Omaha, Nebraska, USA; c Department of Food Science and Technology, University of Nebraska—Lincoln, Lincoln, Nebraska, USA; University of Wisconsin—Madison; University at Buffalo; University of Queensland

**Keywords:** cytokines, dental implants, microbiome

## Abstract

Little is known about longitudinal development of the peri-implant subgingival microbiome and cytokine production as a new sulcus forms after dental implant placement. Therefore, the purpose of this observational study was to evaluate simultaneous longitudinal changes in the oral microbiome and cytokine production in the developing peri-implant sulcus compared to control natural teeth. Four and 12 weeks after implant placement and abutment connection, a dental implant and a natural tooth were sampled in 25 patients for subgingival plaque and gingival crevicular fluid (GCF [around teeth] and peri-implant crevicular fluid [PICF] around implants). DNA from plaque samples was extracted and sequenced using Illumina-based 16S rRNA sequencing. GCF and PICF samples were analyzed using a customized Milliplex human cytokine and chemokine magnetic bead panel. Beta diversity analysis revealed that natural teeth and implants had similar subgingival microbiomes, while teeth had greater alpha diversity than implants. At the genus level, however, few differences were noted between teeth and dental implants over 12 weeks. Specifically, *Actinomyces* and *Selenomonas* were significantly elevated around teeth versus dental implants at both 4 weeks and 12 weeks, while *Corynebacterium* and *Campylobacter* were significantly elevated only at 4 weeks around teeth. The only difference between PICF and GCF biomarkers was significantly elevated granulocyte-macrophage colony-stimulating factor levels around teeth versus dental implants at the 4-week visit. The subgingival microbiome and cytokine production were similar between teeth and implants during early healing, suggesting that these profiles are driven by the patient following dental implant placement and are not determined by anatomical niche.

**IMPORTANCE** Dental implants are a common treatment option offered to patients for tooth replacement. However, little is known regarding initial colonization of the subgingival microbiome and simultaneous longitudinal cytokine production in humans during the early healing phase following implant placement. We report findings from an *in vivo* study that assessed initial colonization of the subgingival microbiome and concomitant early cytokine production in a newly formed anatomical space, namely, an implant sulcus. This approach may be useful in future interventional studies to influence dental implant success. Our data showed that the subgingival microbiome and cytokine profile were similar for control natural teeth and dental implants at both 4 and 12 weeks after implant placement. These data suggest that these profiles are driven by the patient and not by anatomical location (i.e., tooth versus dental implant).

## Abstract (precis)

Dental implants are a common treatment option offered to patients for tooth replacement. However, little is known regarding initial colonization of the subgingival microbiome and simultaneous longitudinal cytokine production in humans during the early healing phase following implant placement. We report findings from an *in vivo* study that assessed initial colonization of the subgingival microbiome and concomitant early cytokine production in a newly formed anatomical space, namely, an implant sulcus. This approach may be useful in future interventional studies to influence dental implant success. Our data showed that the subgingival microbiome and cytokine profile were similar for control natural teeth and dental implants at both 4 and 12 weeks after implant placement. These data suggest that these profiles are driven by the patient and not by anatomical location (i.e., tooth versus dental implant).

## INTRODUCTION

Dental implants are a common option offered to patients for tooth replacement. It is estimated that two to four million implants will be placed into individuals annually in the United States by 2020 (1). Due to the rising trend of implant placement for the management of partially edentulous patients, complications associated with bone loss surrounding dental implants have become more prevalent (1). However, little is known about the initial development and longitudinal progression of the peri-implant subgingival microbiome (2). Past studies examining the initial subgingival colonization patterns following dental implant placement have utilized DNA-DNA hybridization (2, 3) to investigate the differences in select bacterial species in periodontal and peri-implant pockets. Hybridization methods, as well as traditional methods for microbial detection, including bacterial culturing techniques, are hindered by the limited number of bacterial species that can be sampled for testing (4).

Unlike traditional microbial detection methods, next-generation sequencing is a high-throughput method that allows for assessment of ecological diversity and for identification of previously unknown or less abundant bacterial species (5, 6). Previously, multiplexed amplicon pyrosequencing revealed that the microbial peri-implant communities differ from those of natural teeth (7). Specifically, these authors reported that the microbial population within the peri-implant sulcus is less diverse as well as unique from bacterial communities around natural teeth (7).

Next-generation sequencing has previously been used to evaluate the microbiota surrounding natural teeth and dental implants (7, 8). However, to our knowledge, no study has used this technology to evaluate the longitudinal colonization of the subgingival oral microbiome in the newly developed sulcus surrounding recently placed dental implants. Furthermore, the concomitant longitudinal progression of cytokine production after dental implant placement has not been examined. Therefore, the purpose of this observational study was to evaluate longitudinal changes in the oral microbiome and cytokine production in the developing peri-implant sulcus, with neighboring natural teeth as controls, during the first 12 weeks after dental implant placement.

## RESULTS

### Study participants.

**FIG 1:**
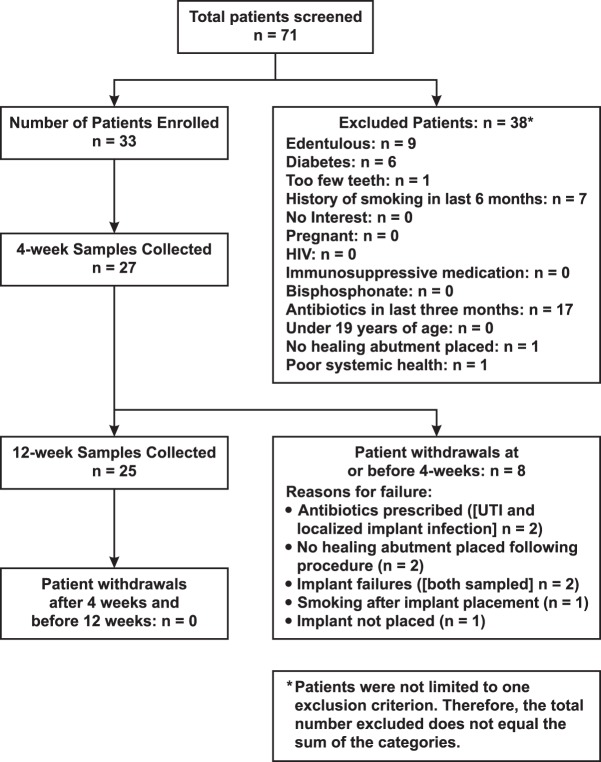
Diagram showing the flow of participants from screening through subgingival plaque and crevicular fluid sample collection at 12 weeks. UTI, urinary tract infection.

The flow of patients from screening through the 12-week visit is shown in Fig. 1. The first patient enrolled in the study on 7 July 2015, and the last patient completed the study on 15 December 2016. Thirty-three patients enrolled in the study, and eight individuals withdrew from the study at or before 4 weeks. Twenty-five participants completed the study and provided subgingival plaque and crevicular fluid samples at both 4- and 12-week visits. Of the 25 participants, only one patient received antibiotic therapy during the study; this patient received a prescription for amoxicillin (500 mg three times daily for 7 days) after the 4-week visit.

Table 1 includes baseline demographic and clinical findings. Participants had a mean age of 60.5 years and were predominantly Caucasian (96%). Nineteen patients had a history of periodontitis. The participants had mean whole-mouth probing depths of 2.5 mm. Bleeding on probing was noted at an average of 5.6% of sites, while supragingival plaque was present at an average of 29.0% of sites. The clinical findings for the control teeth were similar to these whole-mouth data (Table 1).

**TABLE 1:** Baseline sociodemographic data and clinical characteristics of study patients

Baseline sociodemographic factor or clinical characteristic	No. of patients (%) or parameter value
Baseline sociodemographics	
Age (yr) (mean ± SD)	60.5 ± 13.9
Male	14 (56.0)
Female	11 (44.0)
Race	
American Indian or Alaskan native	1 (4.0)
Asian	0
Black or African American	0
White	24 (96.0)
Other	0

Clinical characteristics	
Periodontitis diagnosis	
None	6 (24.0)
Mild	4 (16.0)
Moderate	11 (44.0)
Severe	4 (16.0)
Probing depth (mm) (whole mouth) (mean ± SD)	2.5 ± 0.4
Bleeding on probing (whole mouth) (% of sites)	5.6
Supragingival plaque (whole mouth) (% of sites)	29.0
Estimated probing depth of implants^a^ (mm) (mean ± SD)	1.2 ± 0.5
Probing depth (mm) (control teeth) (mean ± SD)	2.4 ± 0.3
Bleeding on probing (control teeth) (% of sites)	4.0
Supragingival plaque (control teeth) (%)	30.0

^a^ The estimated probing depth of implants is the gingival thickness minus 1.4 mm.

### Subgingival microbiome.

A total of 100 subgingival plaque samples from 25 patients were sequenced, yielding 1.3 million sequences. The number of reads per sample ranged from 25,651 to 70,637 with a mean of 47,821 reads and a standard deviation of 7,769 reads. To standardize sequencing depth, 25,000 reads were subsampled from each file, and rarefaction curves were generated. After the samples were processed, operational taxonomic units (OTUs) were picked as described below in Materials and Methods.

**FIG 2:**
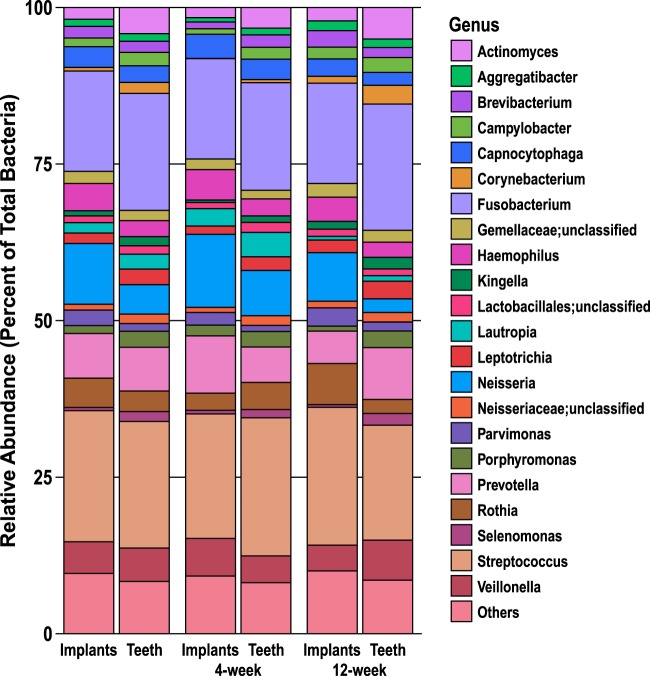
Genus-level composition of subgingival microbiome around implants and teeth for combined 4-week and 12-week subgingival plaque samples (*n* = 50 for each) and for 4-week and 12-week visits separately for implants and teeth (*n* = 25 for each). Genera present at an average relative abundance of >1% are included in the stacked-bar plot and are listed on the right with their respective color coding. Taxa with an average relative abundance of <1% are grouped in “Others.”

Stacked-bar plots were constructed to assess relative abundance at the genus level for taxa with an average relative abundance of >1% (Fig. 2). Those taxa with an average of less than 1% were grouped together in “Others.” The most common taxonomic groups found in the subgingival plaque samples from dental implants at 4 weeks were *Streptococcus* (20%), *Fusobacterium* (16%), *Neisseria* (12%), and *Prevotella* (9%), while the most common taxa in the subgingival plaque samples from dental implants at 12 weeks were *Streptococcus* (22%), *Fusobacterium* (16%), *Neisseria* (8%), and *Rothia* (7%).

Within the subgingival plaque samples from control teeth at 4 weeks, the most common taxonomic groups were *Streptococcus* (22%), *Fusobacterium* (17%), *Neisseria* (7%), and *Prevotella* (6%), while *Fusobacterium* (20%), *Streptococcus* (18%), *Prevotella* (8%), and *Veillonella* (6%) were more common at 12 weeks.

**FIG 3:**
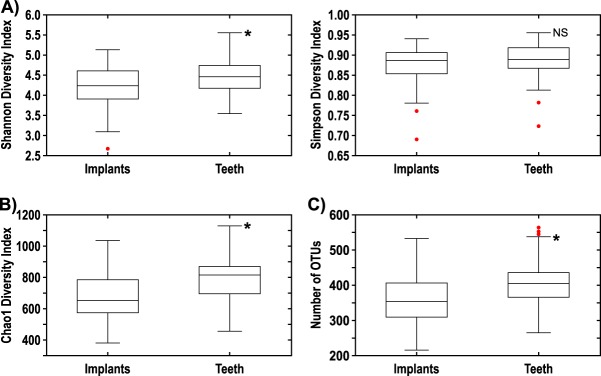
(A) Shannon and Simpson diversity indices comparing implants to teeth for combined 4-week and 12-week subgingival plaque samples. (B) Chao1 diversity index comparing implants to teeth for combined 4-week and 12-week subgingival plaque samples. (C) Number of OTUs comparing implants to teeth for combined 4-week and 12-week subgingival plaque samples. There were 50 samples for both implants and teeth. Values that are significantly different (*P* < 0.01) are indicated by an asterisk. Values that are not significantly different (NS) are indicated.

Alpha diversity measurements (Fig. 3) revealed that control teeth had significantly higher Shannon (*P* = 0.007) and Chao1 (*P* = 0.002) diversity indices and higher number of observed OTUs (*P* = 0.002) than dental implants did. However, the Simpson diversity index did not differ between teeth and dental implants (*P* = 0.127).

**FIG 4:**
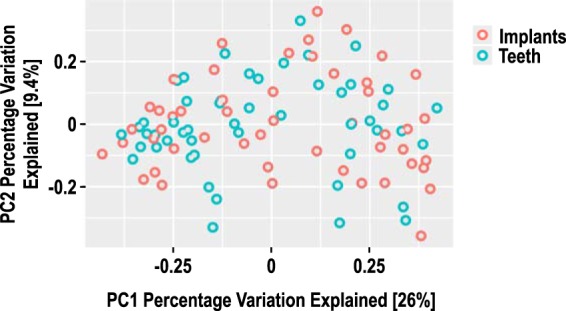
Principal-coordinate analysis of beta diversity based on Bray-Curtis distance for combined 4-week and 12-week subgingival plaque samples (50 samples for both implants and teeth). No evidence of clustering was noted between implants and teeth. PC1, principal coordinate 1.

Principal-coordinate analysis plots (Fig. 4) of beta diversity, based on Bray-Curtis dissimilarity matrix, did not show any obvious clustering between teeth and implants, indicating that these two groups had similar subgingival microbiomes.

**FIG 5:**
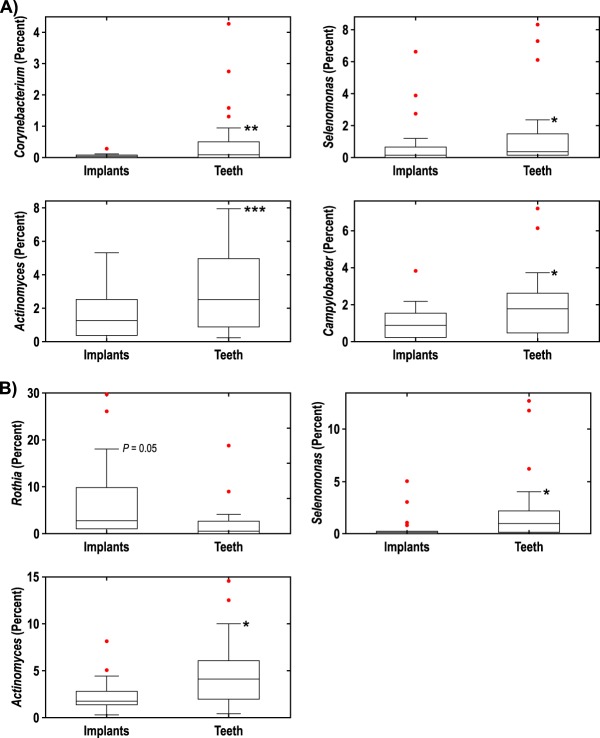
(A and B) Differences at the genus level between teeth (*n* = 25) and implants (*n* = 25) at 4 weeks (A) and 12 weeks (B). Values that are significantly different are indicated by asterisks as follows: *, *P* < 0.05; **, *P* < 0.01; ***, *P* < 0.001.

At the genus level, only a few differences were noted between teeth and dental implants (Fig. 5). At 4 weeks, taxa that were significantly elevated around teeth versus dental implants included *Corynebacterium* (*P* = 0.006), *Actinomyces* (*P* = 0.0007), *Campylobacter* (*P* = 0.0114), and *Selenomonas* (*P* = 0.043). At 12 weeks, the following taxa were significantly elevated around teeth versus dental implants: *Selenomonas* (*P* = 0.046) and *Actinomyces* (*P* = 0.012). *Rothia* appeared to be higher around dental implants relative to control teeth, although the difference was not significant (*P* = 0.0502).

### Cytokines in GCF and PICF samples.

**FIG 6:**
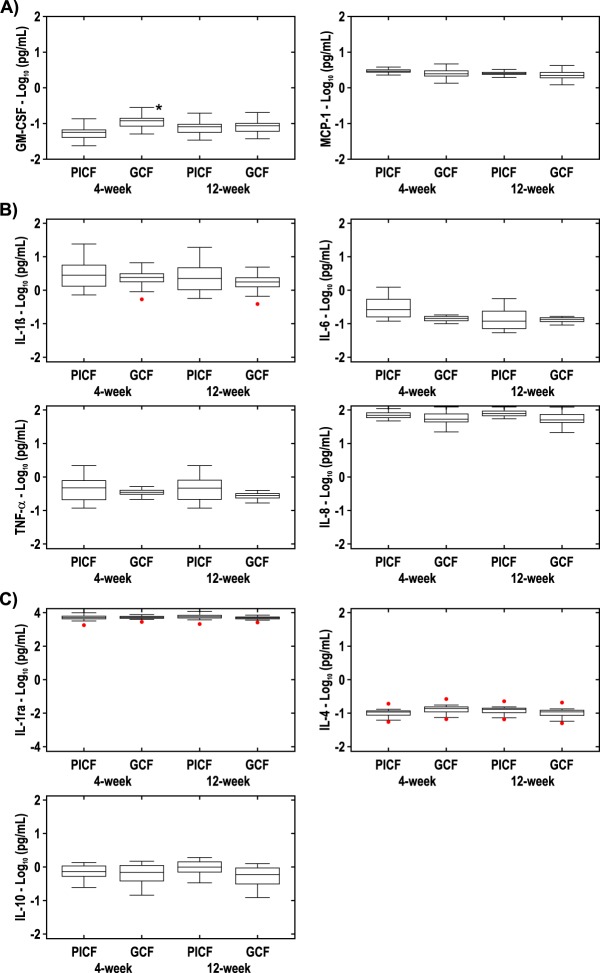
(A) Chemokines in GCF and PICF at 4 weeks (*n* = 25) and 12 weeks (*n* = 25). The GM-CSF value in GCF was statistically significantly different (*P* < 0.01) from the GM-CSF value in PICF as indicated by the asterisk. No other statistically significant differences were noted for the GM-CSF and MCP-1 values between PICF and GCF. (B) Inflammatory cytokines in GCF and PICF at 4 weeks (*n* = 25) and 12 weeks (*n* = 25). No statistically significant differences in these inflammatory cytokine levels were noted between PICF and GCF at either time point. (C) Anti-inflammatory cytokines in GCF and PICF at 4 weeks (*n* = 25) and 12 weeks (*n* = 25). No statistically significant differences were noted between PICF and GCF at either time point.

For each sample, nine cytokines/immune biomarkers were assayed. The results (Fig. 6A) showed that granulocyte-macrophage colony-stimulating factor (GM-CSF) was significantly elevated in gingival crevicular fluid (GCF) at 4 weeks relative to peri-implant crevicular fluid (PICF) (*P* = 0.00786). No other differences were noted between GCF and PICF for any of the other cytokines or chemokines at 4 or 12 weeks (Fig. 6).

### Subgroup analyses for cytokines and subgingival microbiome.

**FIG 7:**
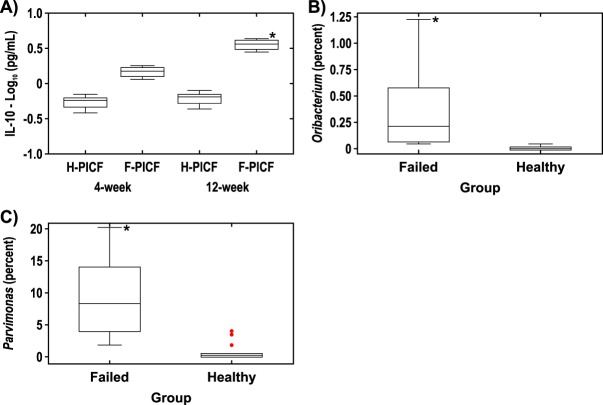
(A) IL-10 in PICF samples from implant sites. PICF samples were taken from healthy implant sites (H-PICF) (*n* = 14) and failed implant sites (F-PICF) (*n* = 4) at 4 weeks and 12 weeks. (B and C) Difference at the genus level at healthy implant sites (*n* = 14) versus failed implant sites (*n* = 4) at 4 weeks (B) and 12 weeks (C). *, *P* < 0.05.

Interleukin-10 (IL-10) was statistically significantly elevated in PICF samples from failed implants (*n* = 4) than in healthy implants (*n* = 14) at 12 weeks (*P* = 0.0346) (Fig. 7A). No other differences in cytokine levels were noted between PICF samples from failed implants versus healthy implants at 4 or 12 weeks (data not shown). No differences in cytokine levels were noted between PICF samples taken from around healthy implants (*n* = 14) versus samples from patients with peri-implant disease (*n* = 11) (data not shown).

At 4 weeks, the only statistically significant difference at the genus level between healthy implants and failed implants was elevated *Oribacterium* around failed implants (*P* = 0.0244) (Fig. 7B), and at 12 weeks, the only statistically significant difference at the genus level between healthy implants and failed implants was elevated *Parvimonas* around failed implants (*P* = 0.0131) (Fig. 7C). No differences at the genus level were noted between healthy implants (*n* = 14) and implants in patients with peri-implant disease (*n* = 11) other than elevated *Oribacterium* at 4 weeks around implants in patients with peri-implant disease (*P* = 0.002); however, the average relative abundance of this organism was less than 0.5% (data not shown).

## DISCUSSION

When a dental implant is placed and an abutment is connected, an open niche is created within the oral environment. Specifically, the new sulcus that forms is initially free of bacteria or, as described by Quirynen et al. (2), a “pristine” pocket is formed. As such, recently placed dental implants provide a unique opportunity to study initial subgingival colonization. Using Illumina-based 16S rRNA sequencing, the current study compared the composition of the subgingival microbiome around newly placed dental implants with that from nearby control teeth. The results showed that the microbiomes in both locations were similar as early as 4 weeks after implant placement. However, two indicators of microbiome diversity, richness and evenness, were decreased around dental implants compared to control teeth. Likewise, cytokine production was generally comparable around newly placed dental implants and control teeth, suggesting that the host responses to the subgingival microbiota were similar at these two sites.

On the basis of relative taxonomic abundance, *Streptococcus* and *Fusobacterium* represented nearly 40% of the microbiome for both implants and control teeth at 4 weeks and 12 weeks. Furthermore, *Prevotella* and *Neisseria* were also among the most common taxa observed both around implants and control teeth. These OTUs were likewise dominant in implant samples, as previously described by Zheng et al. (9) using similar sequencing methods. In contrast, Schaumann et al. (10) reported that *Rothia*, *Streptococcus*, and *Porphyromonas* were the most abundant taxa in the implant subgingival microbiome, while *Prevotella*, *Streptococcus*, and TG5 were the predominant subgingival microbiota around teeth. However, direct comparisons between dental implant microbiome studies are very difficult due to the different microbial analysis methods used, nonstandardized study designs, various patient inclusion/exclusion criteria, and unspecified implant history with respect to when implants were placed prior to the beginning of the study (11).

Dental implants in our study showed lower microbial diversity than control teeth, in agreement with other investigations (7, 12–14). It is possible that the lower microbial diversity around dental implants in our observational study was due to the short-term follow-up after implant placement. However, in each of the above-referenced publications, although the number of years since implant placement was unknown, the implants were not newly placed and the microbial diversity was significantly lower in the subgingival environment around implants than around teeth. Comparing specific taxa between implants and teeth in our study, there were few differences in genera between implants and teeth at either 4 or 12 weeks. At 4 weeks, *Corynebacterium*, *Actinomyces*, *Selenomonas*, and *Campylobacter* were elevated around teeth versus implants. At 12 weeks, only *Actinomyces* and *Selenomonas* were more abundant around teeth. It is possible that, with further maturation of the subgingival microbiota, even these remaining differences would dissipate over time, resulting in further convergence of the microbiomes.

The source of the newly formed subgingival microbiota around dental implants is not well understood. However, saliva is a likely reservoir and conduit to transport microorganisms from the rest of the dentition and from nondental sites such as the tongue, buccal mucosa, and tonsils. It is interesting to note that all study participants rinsed with 0.12% chlorhexidine gluconate twice daily for 1 week following implant surgery. Chlorhexidine gluconate has been shown to significantly reduce supragingival dental plaque formation, even in the absence of brushing (15). However, rinsing with chlorhexidine likely does not result in this medicament reaching the subgingival environment. Furthermore, chlorhexidine gluconate rinsing does not eliminate supragingival plaque formation (15). Therefore, this postoperative regimen does not prevent transfer of microorganisms from other oral and dental sites.

When comparing crevicular fluid cytokines between dental implants and control teeth at 4 and 12 weeks, the overall levels were very similar. However, we noted elevated GM-CSF levels in the crevicular fluid around teeth versus dental implants at 4 weeks. GM-CSF is known to recruit circulating neutrophils, monocytes, and lymphocytes (16). In the current study, control teeth sites were scaled subgingivally at the baseline dental implant placement appointment. The patients in our study refrained from brushing the newly placed dental implants and control teeth for 1 week, although the participants used, as mentioned above, a plaque-reducing mouth rinse during that time frame. It is quite likely that recolonization of the subgingival microbiota at these control tooth sites triggered a localized host response, resulting in elevated GM-CSF to recruit inflammatory cells.

There are few published studies assessing GM-CSF formation around dental implants. A recently published experimental gingivitis study examined cytokines, including GM-CSF, around existing implants which had been present for at least 1 year in patients who had refrained from brushing both dental implants and teeth for 3 weeks (17). At day 21, IL-1β, GM-CSF, tumor necrosis factor alpha (TNF-α), and interferon gamma (IFN-γ) were significantly higher in GCF than PICF. Although in our study, we did not observe differences in IL-1β and TNF-α between GCF and PICF samples, the patient populations and study designs were different in the studies, as noted above. It is possible that no other cytokine or chemokine differences were observed for dental implants and control teeth in our study due to the timing or frequency of crevicular fluid sample collection, as our sampling may have missed transient increases or decreases in cytokine levels.

Although IL-10 is primarily recognized for its anti-inflammatory properties, this cytokine can have either favorable or unfavorable effects on the host depending on the nature of the bacterial infection or the levels of IL-10 produced (18). In subgroup analyses, IL-10 was elevated in failed implants versus healthy implants at 12 weeks, in agreement with Ata-Ali et al. (19). In contrast, Casado et al. (20) showed that IL-10 concentrations were lower in patients with peri-implantitis than in patients with healthy implants, while other studies have reported no differences (21, 22). The findings in our study suggest that elevated IL-10 reflects an attempt by the host to limit the development of an inflammatory response around failed implants.

When the microbiota around failed implants was examined, we observed that *Parvimonas*, a genus that includes the periodontal pathogen Parvimonas micra, and *Oribacterium*, a genus not associated with periodontitis (23), were elevated relative to healthy implants. Koyanagi et al. (8) and Tamura et al. (24) similarly reported that *P. micra*, a member of the orange complex (25), was detected in patients with peri-implantitis. The microbiome and cytokine findings in our subgroup analyses should be interpreted with caution due to the small sample sizes of each subgroup and the exploratory nature of these analyses.

One limitation of this study was the short duration. The study duration was only 12 weeks because the plan was for patients to proceed with implant restoration shortly thereafter, which would have likely influenced the subgingival microenvironment. However, regarding fluctuations in the oral microbiome over time, recent evidence indicates that 83% of oral microbial samples taken a year after initial sampling could be confidently traced back to the source subject; indeed, phylogenetic diversity remained relatively stable over time within individuals over 1 year compared to interindividual variation (26). However, strengths of our study include evaluation of the entire subgingival microbiome rather than targeted organisms and simultaneous evaluation of a panel of cytokines. Indeed, to our knowledge, this is the first study to examine both the subgingival microbiota and host response during early dental implant healing. In addition, we propose that this novel experimental approach can be used as a human *in vivo* model for other investigations of initial subgingival colonization and early cytokine production in a newly formed anatomical space, namely, an implant sulcus.

The mouth is easily accessible for simultaneous collection of subgingival plaque samples for microbial analysis and crevicular fluid samples for cytokine detection around dental implants. With this approach, initial subgingival colonization and cytokine production can be studied in patients with various systemic conditions (e.g., poorly controlled diabetes and rheumatoid arthritis) which have been shown to impact periodontal (27, 28) and implant (29, 30) health and in smokers, as smoking is a major environmental risk factor for both periodontitis (31) and implant failure (32). Furthermore, this model can be used to study the impact of pharmaceutical interventions to potentially alter initial subgingival microbiome formation and early cytokine production and, thereby, possibly influence implant success.

In summary, in this 12-week observational study, the subgingival microbiome and cytokine production were similar in teeth and dental implants during early implant healing. These data suggest that initial subgingival microbial colonization and cytokine production are driven by the patient and are not determined by anatomical niche (i.e., implant versus tooth). Furthermore, future studies can potentially use this human *in vivo* model to examine initial subgingival microbiota development and simultaneous cytokine production following dental implant placement in patient populations with various systemic diseases and environmental risk factors for implant failure.

## MATERIALS AND METHODS

### Study participants.

Approval for this observational study was obtained from the University of Nebraska Medical Center (UNMC) Institutional Review Board (protocol number 202-15-EP). Partially edentulous patients were enrolled from the UNMC College of Dentistry’s Department of Surgical Specialties, Section of Periodontics. Twenty-five patients completed the 12-week study and provided both subgingival plaque and crevicular fluid samples from a recently placed dental implant and a control tooth.

Patients in good general health receiving at least one dental implant, including the placement of a transmucosal healing abutment, were potentially eligible for this study. Exclusion criteria for study enrollment were as follows: (i) pregnancy; (ii) diabetes; (iii) HIV infection; (iv) currently taking systemic immunosuppressant medications; (v) currently taking any bisphosphonates; (vi) antibiotics in the previous 3 months; (vii) patient self-report of smoking in the previous 6 months; (viii) requiring preoperative prophylactic antibiotics; (ix) fewer than 15 teeth present; (x) under 19 years of age. All participants provided written informed consent before study enrollment.

Individuals enrolled in this study had already decided to pursue implant treatment prior to being approached regarding study participation. All patients participated in the College of Dentistry’s implant screening and planning process. Patients received a comprehensive periodontal examination and were screened for dental implant placement by a periodontal resident. The patient’s periodontitis case definition was determined based on measurements of probing depths and clinical attachment loss according to Eke et al. (33). Bleeding on probing and plaque score were also recorded. A prosthodontist then evaluated the restorability of edentulous sites with dental implants. Finally, members of the restorative and surgical team met prior to the placement of the dental implant to discuss any relevant treatment considerations. These considerations included systemic patient health factors, restorative considerations, and periodontal health considerations. No patients in this study required active periodontal treatment.

### Day of implant placement.

Patients were screened for study inclusion on the day of implant placement on a rolling admission basis. Medical history was reviewed prior to each implant procedure. Sociodemographic data were collected and included age, gender, race, and ethnicity. All implants were placed at posterior sites (premolar or molar) with the exception of one implant placed at an anterior (cuspid) location. A tooth was selected for evaluation as a control within the same quadrant as the implant site. The control tooth was the first available tooth mesial to the implant site and was separated from the implant by at least one natural tooth in order to avoid possible influence from the adjacent developing implant sulcus (34).

Probing depths, bleeding on probing, and supragingival plaque were recorded for the control tooth. Subgingival scaling was completed presurgically on the control tooth chosen to be sampled. During the implant surgery, flaps were reflected, and gingival thickness was measured from six different sites surrounding the implant with a Marquis probe (Hu-Friedy, Inc., Chicago, IL) (2). A transmucosal healing abutment was used for all patients. Implants were manufactured by Nobel Biocare (Yorba Linda, CA); root form, tapered implants were placed. Patients were instructed to avoid brushing at or near the surgical site for 1 week. All patients were prescribed and instructed to gently rinse with 0.12% chlorhexidine gluconate for 30 s twice per day for 1 week (2). Postoperative acetaminophen or hydrocodone with acetaminophen was advised for postoperative analgesia.

### Four-week GCF, PICF, and subgingival plaque sample collection.

Medical history and exclusion criteria were reviewed for patients returning for the 4-week postoperative and sample collection visit. All patients were assessed for any signs of infection or failure of implant osseointegration at this appointment.

Crevicular fluid samples, followed by subgingival plaque samples, were collected at the dental implant and control tooth sites on patients who continued to be eligible for study participation. Prior to crevicular fluid and subgingival plaque sample collection, supragingival plaque was removed around the implant abutment and control tooth by scaling. The implant and control tooth were isolated using cotton rolls and gently air dried. Crevicular fluid samples were then collected by inserting filter paper strips (Periopaper; Proflow, Amityville, NY), until slight resistance was felt, into the mesiobuccal and mesiolingual peri-implant and tooth sulci for 30 s. The two peri-implant crevicular fluid (PICF) samples for the implant were placed into a single microcentrifuge tube on ice beside the dentist’s chair and frozen at −80°C within 10 min of collection. Likewise, the same procedure was followed for the two gingival crevicular fluid (GCF) samples taken from the control tooth.

Peri-implant plaque samples were collected by inserting four separate sterile endodontic paper points (Henry Schein medium absorbent points; Melville, NY) into the mesial, distal, buccal, and lingual sites (i.e., circumferentially around the implant) for a period of 10 s each. Subgingival plaque samples collected from around the dental implant were pooled in a single microcentrifuge tube and frozen at −80°C within 10 min of collection. The same procedure was followed for the plaque samples taken from the control tooth. GCF, PICF, and subgingival plaque samples remained frozen at −80°C until analysis.

### Twelve-week GCF, PICF, and subgingival plaque sample collection.

Twelve weeks after the initial implant placement, patients returned, and the same protocol was performed as described above for the 4-week sample collection appointment. All plaque, GCF, and PICF samples at both 4-week and 12-week visits were collected by the same investigator (P. G. Johnson).

### DNA extraction of plaque samples.

DNA extraction was performed by the method of Martinez et al. (35) with slight modifications. Briefly, 200 µl of ice-cold phosphate-buffered saline (PBS) was added to tubes containing the paper points and the tubes were vortexed for 15 min. Next, 750 μl of lysis buffer and 20 mg/ml of lysozyme were added to the tubes, and the samples were then transferred into bead beating tubes containing 300 mg of disruption beads (0.1 mm; Research Products International Corp., Mount Prospect, IL). The samples were incubated for 15 min in a 37°C water bath. Then, 85 μl of 10% SDS solution and 40 µl of proteinase K (15 mg/ml) were added to the tubes, and the samples were incubated for 15 min in a 60°C water bath. Five hundred microliters of phenol-chloroform-isoamyl alcohol (25:24:1) was added to each tube, and the samples were homogenized using a mini-bead beater (BioSpec Products, Bartlesville, OK) for 2 min at high speed. The samples were then centrifuged at 10,000 × *g* for 5 min, and the top aqueous layer was extracted. This extraction was repeated twice using 500 μl of phenol-chloroform-isoamyl alcohol (25:24:1) and twice using 500 μl chloroform-isoamyl alcohol (24:1). Overnight ethanol precipitation was used to recover DNA, and the DNA pellets were dissolved in 100-μl portions of Tris-HCl buffer (10 mM; pH 8.0). DNA was quantified using a NanoDrop ND-1000 spectrophotometer (NanoDrop Technologies, Inc., Wilmington, DE) and stored at −20°C.

### Sequencing and microbial community analysis.

The DNA samples were submitted to Neogen GeneSeek Operations (Lincoln, NE) for 16S rRNA paired-end sequencing using the Illumina MiSeq system. The primers used were 515F (F stands for forward) (5′-GTGCCAGCMGCCGCGGTAA-3′) and 806R (R stands for reverse) (5′-GGACTACHVGGGTWTCTAAT-3′), flanking the 515 and 806 region. Barcodes were attached to the 806R primers, and 2 × 250 reads were sequenced on the Illumina MiSeq system. Quantitative Insights into Microbial Ecology (QIIME) was used to analyze the microbial community (36). Briefly, sequences were demultiplexed, and barcodes were removed prior to analysis. The sequences were checked for quality using FastQC (http://www.bioinformatics.babraham.ac.uk/projects/fastqc/) and sequences with a phred score of less than 20 were trimmed with TrimGalore (https://www.bioinformatics.babraham.ac.uk/projects/trim_galore/). Sequences with a maximum length of 255 bp were merged using PANDAseq (37) with a threshold score of 0.9. Reads with ambiguous nucleotides were removed during the merge. Using subsampled open reference operational taxonomic unit (OTU) picking in QIIME (38), the sequences were first referenced at a 97% similarity against the Human Oral Microbiome Database (HOMD 16S rRNA RefSeq V13.2). Sequences that did not hit the database were clustered into OTUs at a 97% sequence similarity using Uclust, followed by PyNAST alignment. Taxonomy was assigned using QIIME’s Uclust-based Consensus Taxonomy Assigner. In QIIME, ChimeraSlayer was used to check for chimeric sequences after OTU picking. QIIME was subsequently used to analyze both alpha and beta diversities of the oral microbial community. Specifically, Shannon index, Simpson index, Chao1 richness estimator, and the number of observed OTUs were used to determine alpha diversity, while beta diversity was visualized using principal-component analysis (PCoA) based on Bray-Curtis distance (36).

### GCF and PICF cytokine determination.

At the time of analysis, each pooled GCF and PICF sample was eluted using 60 μl of 1× PBS by gently agitating the samples on a rocker plate for 1 h at 4°C. Aliquots for interleukin-1 receptor antagonist (IL-1ra) assessment were diluted at 1:100 so that the cytokine levels were not above the upper limit of the standard curve. Aliquots to determine the amounts of the other cytokines present in GCF or PICF were not diluted. Cytokine and chemokine concentrations were measured using a customized human cytokine and chemokine magnetic bead panel (Milliplex MAP kit; Millipore, Billerica, MA), and a MAGPIX instrument and software (Luminex Corporation, Austin, TX) per the manufacturers’ recommendations. Nine analytes were measured: interleukin-1β (IL-1β), IL-1 receptor antagonist (IL-1ra), IL-4, IL-6, IL-8, IL-10, granulocyte-macrophage colony-stimulating factor (GM-CSF), monocyte chemoattractant protein 1 (MCP-1), and tumor necrosis factor alpha (TNF-α). The amounts of cytokines are reported in picograms per 30-s sample.

### Statistical analyses. (i) Subgingival microbiome.

Statistical analyses were performed using R version 3.3.2 (R Core Team, 2016, R Foundation for Statistical Computing, Vienna, Austria), QIIME, linear discriminant analysis effect size (LEfSe), and analysis of composition of microbiomes (ANCOM) to compare control teeth (*n* = 25) and dental implants (*n* = 25) at 4 and 12 weeks. In QIIME, *P* values were obtained from nonparametric two-sample *t* tests using Monte-Carlo permutations to compare alpha diversity measures of control teeth and dental implants.

In R, nonparametric significance tests, permutational multivariate analysis of variance (PERMANOVA), and analysis of similarities (ANOSIM) based on Bray-Curtis dissimilarity coefficients were used to compare community composition between groups. Differential taxonomic groups between control teeth and dental implants were identified using four differential abundance analysis models: RF (random forest) and classification tree analysis in R, LEfSe to compute linear discriminant analysis (LDA) effect size (39), and ANCOM (40). Top hits were identified as the taxa that were successfully differentiated in at least two models, and the relative abundance of the top hits was compared between control teeth and implants using the pairwise Wilcoxon signed-rank test. The differences in taxa between the groups were considered significant at *P* < 0.05 after Bonferroni’s correction.

### (ii) GCF and PICF cytokine determination.

All statistical analyses were conducted using the R software, version 3.3.2 (R Core Team, 2016, R Foundation for Statistical Computing, Vienna, Austria). Statistical modeling was done using the lmerTest package using the lmer function (41). Comparisons were made between the control teeth (*n* = 25) and dental implants (*n* = 25) at 4 and 12 weeks.

Cytokine concentrations were log transformed. Then, comparisons between PICF and GCF were performed using a generalized linear mixed-model approach that included a parametric analysis of variance (ANOVA) (i.e., log_10_ transformed cytokine concentration) with the group (implant versus control natural tooth) as the fixed effect and the site by patient as the random intercept. Specifically, comparisons between PICF versus GCF over time were performed using the group [i.e., interaction between two anatomical sites for each of the 25 patients with two time points, 4 and 12 weeks] as the fixed effect and the site by patient as the random effect [i.e., this term assumed that the effect of patient is random and that both the intercept and slope of site depended on each patient]. Thereafter, all posthoc testing was done using Tukey’s test adjusted for multiple comparisons using Bonferroni’s correction. Significant differences across groups were considered if *P* < 0.05.

### (iii) Subgroup analyses.

Of the 25 implants that were sampled 4 weeks and 12 weeks after placement, 14 were entirely healthy at 12 weeks (i.e., no evidence of inflammation or alveolar bone loss), while 11 implants had signs of peri-implant disease (evidence of inflammation or alveolar bone loss), four of which failed at 12 weeks and were removed.

The following subgroup analyses were performed with respect to the subgingival microbiome and crevicular fluid: healthy implants (*n* = 14) versus implants with evidence of peri-implant disease (*n* = 11) and healthy implants (*n* = 14) versus failed implants (*n* = 4). For the subgingival microbiome, the same statistical analyses as outlined above, for the entire study population, were performed with the exception of using the unpaired Wilcoxon test to compare the top differential taxa of the subgroup comparisons. Crevicular fluid cytokine and chemokine comparison for healthy implants (*n* = 14) and implants that failed at 12 weeks (*n* = 4) and comparison between healthy implants and implants with evidence of peri-implant disease (*n* = 11) were done via a linear mixed-model approach using the group as the fixed effect and the patient as the random effect (i.e., random intercept by patient).

### Accession number(s).

Sequences have been deposited in the NCBI database under BioProject accession number PRJNA417321.
